# Two-year scale-up dissemination study of a multi-strategic community-wide intervention promoting physical activity: a single-arm pre-post hybrid effectiveness-implementation trial

**DOI:** 10.1186/s12966-024-01684-6

**Published:** 2024-11-25

**Authors:** Aoi Tsuzuki, Masamitsu Kamada, Shiho Amagasa, Jun Kitayuguchi, Takuma Miyashita, Takafumi Abe, Tatsunosuke Gomi, Kenta Okuyama, Masataka Taguri, Shigeru Inoue

**Affiliations:** 1 Department of Health Education and Health Sociology, School of Public Health, Graduate School of Medicine, The University of Tokyo, 7-3-1 Hongo, Bunkyo-Ku, Tokyo, 113-0033 Japan; 2 Graduate School of Public Health, Teikyo University, Itabashi-Ku, Tokyo, Japan; 3 Department of Preventive Medicine and Public Health, Tokyo Medical University, Shinjuku-Ku, Tokyo, Japan; 4 Physical Education and Medicine Research Center UNNAN, Unnan, Shimane, Japan; 5 Center for Community- Based Healthcare Research and Education (CoHRE), Shimane University, Izumo, Shimane, Japan; 6 Research Team for Promoting Independence and Mental Health, Tokyo Metropolitan Institute for Geriatrics and Gerontology, Itabashi-Ku, Tokyo, Japan; 7 Center for Primary Health Care Research, Lund University, Malmö, Sweden; 8 Department of Health Data Science, Tokyo Medical University, Shinjuku-Ku, Tokyo, Japan

**Keywords:** Exercise, Resistance training, Scale-up, Dissemination, RE-AIM

## Abstract

**Background:**

Intervention trials that have demonstrated significant effects may not always replicate those effects when scaled up. This study aimed to test whether scaling-up a successful cluster randomized trial (the COMMUNICATE study, 9 intervention communities), which promoted population-level physical activity (PA), could promote PA in a broader citywide setting (29 communities) after two years, as a mid-term evaluation of the six-year scaled-up trial.

**Methods:**

This is a single-arm, pre-post comparison of a multi-strategic community-wide intervention covering the entire Unnan City, Japan. The intervention for middle-aged and older people consisted of three components: information delivery, education, and support delivery. The intervention method followed the COMMUNICATE study but adapted and introduced new initiatives tailored to local resources. A baseline survey (*n* = 3,718) among randomly selected residents aged 40–79 years in 2016 and a follow-up survey with the same respondents two years later were conducted. The primary outcome was the change in the percentage of people who practiced the recommended levels of PA, analyzed with a generalized linear mixed model to account for clusters at individual and community levels. Additionally, we examined the dose–response relation of the effect based on the intervention doses in each community. The RE-AIM framework assessed intervention dissemination and implementation.

**Results:**

The two-year intervention was implemented in all communities, reaching and involving various groups. The rate of people engaged in PA significantly increased in two years (adjusted change: + 8.0 percentage points [95% confidence interval: 6.1, 10.0]). Based on the type of PA, only muscle-strengthening activity showed a significant increase (+ 11.5% points [9.6, 13.5]), whereas walking (-1.8% points [-3.6, 0.1]) and flexibility activities (+ 0.3% points [-1.5, 2.0]) did not. The increase in PA in higher-dose areas was not significantly different but slightly larger than that in lower-dose areas (+ 8.4% points vs. + 7.6% points, adjusted difference in change: 0.8% points [-3.8, 5.5]).

**Conclusion:**

The scaled-up citywide intervention promoted PA, especially muscle-strengthening activity. Collaboration with diverse organizations in different settings is crucial for multi-faceted interventions and requires balancing uncertainty in its implementation quality and quantity owing to collaborative decision-making.

**Trial registration:**

UMIN-CTR, UMIN000024682. Registered 02 November 2016, https://center6.umin.ac.jp/cgi-open-bin/ctr/ctr_view.cgi?recptno=R000028377

**Supplementary Information:**

The online version contains supplementary material available at 10.1186/s12966-024-01684-6.

## Introduction

Although physical activity has been recognized for its positive effects on health [1], physical inactivity is highly prevalent worldwide [2, 3]. The World Health Organization has set a goal of a 15% relative reduction in the global prevalence of physical inactivity among adults by 2030 and has proposed several policies, one of which is the implementation of community-wide or whole-of-community initiatives [4]. Community-wide programs represent interventions that appeal to the entire community at multiple levels with the goal of encouraging behavioral changes [4–6]. While this intervention is theoretically recommended by ecological models [7, 8], evidence supporting its benefits is limited [9]; a 2015 Cochrane review identified 33 community-wide intervention studies and reported that few studies with low risk of bias showed effectiveness in promoting physical activity, making it difficult to draw conclusions on effectiveness [6]. More recent studies have reported that long-term community-wide programs increased physical activity at the community level [10, 11]. The COMMUNIty-wide CAmpaign To promote Exercise (COMMUNICATE) study, a five-year cluster randomized controlled trial (cRCT) conducted in Unnan City, Shimane Prefecture, Japan, employed social marketing techniques and demonstrated an increase in population-level physical activity among middle-aged and older adults [10].

Scale-up interventions are required to address global physical inactivity [12]. However, it is unknown whether intervention trials that achieved significant benefits will have the same effects when expanded to wider communities and societies in a real-world context. The evaluation of the scalability of intervention trials is necessary, but the evidence, again, remains limited [12–16]. A systematic review comparing the effects of RCTs and scale-up interventions to promote physical activity identified eight pairs of RCTs and scale-up interventions; however, while some showed an increase in the effect owing to scaling up, others showed that the effect dwindled, and the amount of change reported also varied [16]. Nevertheless, the review did not include community-wide programs promoting the physical activity levels of the entire community. Moreover, the scale-up interventions identified in the review were short, lasting only from 10 weeks to 12 months. This short duration increased the difficulty of examining the sustainability of the interventions and their effects.

Therefore, the present study aimed to clarify the effectiveness and implementation of a scale-up intervention of the community-wide program, which was shown to be effective in promoting physical activity in a cRCT (the COMMUNICATE study) [10], on the population-level physical activity over a two-year period. The duration of the scale-up intervention was scheduled to be six years, extended by one year from the originally planned five-year period owing to the torrential rain disaster in 2021. The current work evaluated the effects two years after the start of the program.

## Methods

This study reports the two-year evaluation of the scale-up (2016–2018) of the preceding cRCT (2009–2014), which was effective in promoting physical activity among middle-aged and older adults [10]. The study design is a single-arm study involving pre-post evaluations and began in November 2016. It is a hybrid type II effectiveness-implementation trial, which includes implementation evaluations in real-world situations [17]. Most of the evaluations followed the methods used in the preceding cRCT [10]. As an additional post-hoc quasi-experimental element, it also included dose–response analysis based on the intervention doses that naturally differ across communities, aimed at strengthening our causal inference. The study location was Unnan City (population 40,372 and area 553.17 km^2^ as of 2016), Shimane Prefecture, Japan. This study was registered with UMIN-CTR (UMIN000024682) and approved by the Research Ethics Committee of the Physical Education and Medicine Research Center UNNAN.

**Fig. 1:**
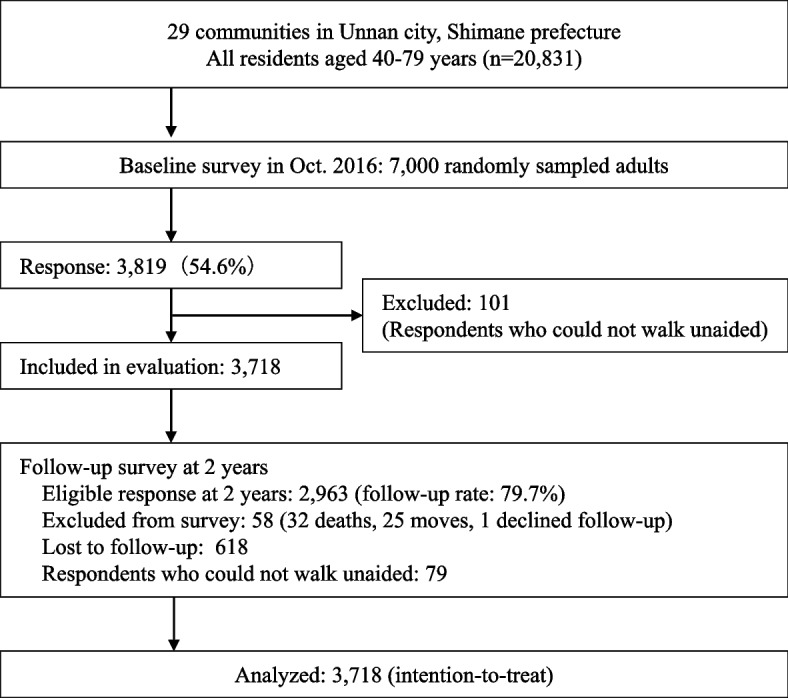
Flowchart of the study

Figure 1 shows the study outline and participant flow. The present scale-up intervention covered the entire Unnan City, which consists of 30 communities (equivalent to elementary school districts) while the former cRCT was implemented in 12 randomly selected communities. Following the methodology of the preceding cRCT, which considered the units of residents’ (communities’) social activities, this scale-up study regarded *Yokaichi* and *Sanshinto* as the same community in the analysis. Thus, the study analyzed a total of 29 communities.

### Intervention

The intervention was a multi-strategic community-wide program led by the local government of Unnan City (responsible department: Physical Education and Medicine Research Center UNNAN). Details of the intervention providers (members of the department) are described in the Appendix Text. The scale-up intervention began in November 2016 targeting all residents aged 40–79 years in Unnan City. In the two years to 2018, meetings with various stakeholders, such as public health nurses working for the city hall and resident volunteers, were held to build collaborative networks across the city. The present scale-up trial continued the basic promotion strategies developed through social marketing and logic model [18] during the cRCT period; that is, the intervention was composed of 1) information delivery, 2) education, and 3) support delivery. The intervention delivery units and providers were flexible, with the local government of Unnan City offering support and expertise to collaborators where necessary. However, the targeting approach was somewhat relaxed, rather than strictly defining the priority audience as women aged 60–79 years with low back pain and insufficient physical activity at baseline [18]. This adjustment allowed for the inclusion of other segments of middle-aged and older residents, and intervention adaptations were established. The major adaptations of each component were as follows; 1) utilization of citywide cable television and city newsletters for information delivery, 2) initiation of a citywide initiative of a peer-led muscle-strengthening program, the Unnan *Kou-un* (Happiness in Unnan) Exercise, and 3) trained volunteers as community exercise leaders in all communities; for a new physical environmental support, an existing swimming pool facility was restored and opened to all residents as the city’s core health promotion center. Details of each component and adaptation process are described in the Appendix Text and Appendix Table 4.

### Implementation evaluation

The implementation status was assessed based on the records from the local government of Unnan City and collaborators. We examined (1) the utilization of distributed documents and media for information delivery, (2) the documentation of health education programs and Unnan *Kou-un* Exercise for education, and (3) the activity levels of community exercise leaders and utilization of the health promotion center for support delivery.

The overall intervention was evaluated using the RE-AIM framework, utilizing these quantitative records. RE-AIM is a framework for assessing the impact of interventions on public health, considering five key dimensions: *Reach*, *Effectiveness*, *Adoption*, *Implementation*, and *Maintenance* [19]. In this study, *Reach* was determined by assessing the percentage of the population covered by distributed information materials and the quasi-population coverage rate of participants in health education programs (i.e., gross number of participants/population aged 40–79 years). *Effectiveness* was evaluated through the statistical analyses detailed in the section below. *Adoption* was determined by the percentage of communities that adopted the intervention. *Implementation* was assessed using the global implementation score across the entire city. The score was calculated by researchers who were not involved in the scale-up intervention (AT and MK), referring to the implementation scoring method from previous studies [10, 20] and subsequently validated through discussions with the intervention implementers (Unnan City personnel, including JK and TM). Appendix Table 1 presents the criterion for the implementation score. *Maintenance* was evaluated based on the status of promoting physical activity in 2022 (maintenance at the organizational level). Meanwhile, *Maintenance* of behavioral changes at the individual level is a future objective for a planned study.

### Population-based evaluation

The quantitative outcomes regarding the intervention effectiveness were all measured by population-based surveys designed as a representative cohort study. For the baseline survey, self-administered questionnaires were sent to 7,000 residents randomly selected by a computer-based resident registry system in November 2016 (see statistical analysis section below for sample size calculation). Respondents aged 40–79 years who lived in Unnan City were eligible; individuals residing in facilities and individuals requiring long-term care or support were excluded. Written informed consent was obtained from all the respondents. The analysis excluded those who required walking assistance. A two-year post-survey was conducted in November 2018 as a follow-up of all the participants who completed the baseline survey. The survey was conducted by the local government of Unnan City, and the statistical analysis was performed at the University of Tokyo (AT and MK) using de-identified data.

### Measures

The primary outcome was the citywide change in engagement in regular physical activity, measured at the individual level at baseline and two-year follow-up. Individuals who met one or more of the following three conditions were defined to be performing regular physical activity: (1) ≥ 150 min/week of walking, (2) daily flexibility activity, or (3) ≥ 2 days/week of muscle-strengthening activity. These three conditions were based on the guidelines [21–23] and were identical to the definitions of physical activity used in the cRCT for longer-term observation [10]. Flexibility activities are recommended for older adults, particularly those with musculoskeletal disorders [22–24]. In Japan, musculoskeletal disorders have placed a significant burden on both individuals and society [25]. The cRCT anticipated the positive effects of flexibility activities when promoting physical activity, and this approach was carried forward in the current scale-up.

Regarding walking time, the respondents were asked about the number of days a week they walked, and the mean number of minutes they spent walking daily, whether for recreational or transportation purposes. We calculated the total amount of time spent walking per week. The flexibility activity was assessed categorically (daily, not daily but occasionally, rarely). The participants also reported the number of days per week they performed muscle-strengthening activity. These questions were identical to those used in the cRCT. The test–retest reliability of the walking time for a 10-day interval and criterion-related validity using an accelerometer were both acceptable (Spearman’s *r* = 0.79 and 0.38, respectively) [18]. The test–retest reliability of the flexibility and muscle-strengthening activities were also within permissible range (weighted kappa = 0.72 for flexibility and Spearman’s *r* = 0.75 for muscle-strengthening activity) [18].

As covariates, data on the body mass index (BMI, kg/m^2^), self-rated health (very good, good, not very good, not good), years of education, engagement in paid work, engagement in farming, and history of chronic disease were collected at the baseline survey, and data on sex, age and community of residence were obtained from the residential registry. Additionally, information on adverse events (harms) during the educational programs was obtained from the regular reports provided by the local government of Unnan City.

### Statistical analysis

The sample size of 7,000 was calculated based on the data obtained from the cRCT; a two-sided significance level of 5% and a power of 90%, assuming a 60% response rate and a 4% increase in engagement in regular physical activity in the entire city, considering community-level clustering with an intracluster correlation coefficient (ICC) of 0.0032 [10]. A multilevel analysis was performed as the primary analysis. The change in the primary outcome of regular physical activity was estimated by a generalized linear mixed model (GLMM) with sex, age, BMI, self-rated health, education, employment, farming, history of chronic disease, and a community where respondents lived as fixed effects, and individuals as a random effect. Community was included as a fixed effect to partial out all the observed and unobserved time-invariant community-level confounding factors [26].

We also conducted subgroup analyses after stratification by each covariate and intervention status (community-level assignment, i.e., intervention, control, or excluded) in the cRCT, using similar GLMM. Intervention status in the cRCT was also used to determine if the effect of the cRCT-intervention (2009–2014) continued using the data in 2016 as a cross-sectional study.

As post hoc analysis, we verified the dose–response relation between the amount of intervention and the change in engagement of regular physical activity. This test was based on the hypothesis that communities with higher amounts of intervention would have a greater increase in the proportion of people engaging in physical activity, which could support the causal inference for the intervention’s effect on the primary outcome. It is important to note that efforts were made to implement the intervention citywide in a way that minimized differences between communities. Based on the implementation score, which represents naturally differed intervention doses across communities (details in Appendix Text and Appendix Tables 1 and 2), the communities were divided into high and low dose groups, ensuring roughly equal sample sizes. The community implementation score was calculated using two education-related items and two items related to support delivery, excluding items related to information delivery, which exhibited relatively minor differences across the communities.

All analyses were on an intention-to-treat basis and included all baseline respondents who could walk unaided. Missing information was imputed to minimize bias and repeated nine times. Ten imputed datasets were created, assuming missing at random, using regression models with analysis variables. Each dataset was independently analyzed and combined for inference. To examine for any attrition bias associated with loss of follow-up in the evaluation cohort, we noted the proportion of those with missing data on the physical activity outcomes in the two-year follow-up survey. All analyses were performed using SAS version 9.4 (SAS Institute Inc., Cary, NC, USA) with the statistical significance set at 5%.

## Results

The overall intervention was evaluated using the RE-AIM framework, utilizing quantitative records from the intervention providers (Appendix Fig. 2). For *Adoption*, all 29 communities participated in the project. As for *Reach* for information, 100% was achieved because the Unnan City government published 10 articles about physical activity in the city newsletter, which was distributed to all households in the city. However, the second measure of *Reach*, representing the quasi-population coverage of educational opportunities, was 10.6%. Appendix Tables 2 and 3 list the *Implementations*, the dose of the intervention implemented citywide, as well as classification by group based on the implementation score. The global implementation score of this scale-up intervention was lower than that in the cRCT phase (78 vs 94). Despite the comprehensive implementation of all the three intervention components, the dose varied among the communities (Appendix Tables 2 and 3). For support delivery, the community exercise leaders significantly contributed to disseminating knowledge through conversation and supporting exercise classes, with these efforts increasing over two years (e.g., the number of exercise classes they involved: 442 times in 2017 and 935 in 2018). The Unnan City government aims to train leaders for all the communities, with only two communities lacking leaders as of 2018. The scale-up intervention, including all of three components (i.e., information delivery, education, and support delivery), continued through 2022 as planned, indicating organization level *Maintenance* as 100%. Two adverse events (harms: 1 muscle spasm; 1 fall without injury) occurred during the educational program, but no serious injuries were reported.

**Fig. 2:**
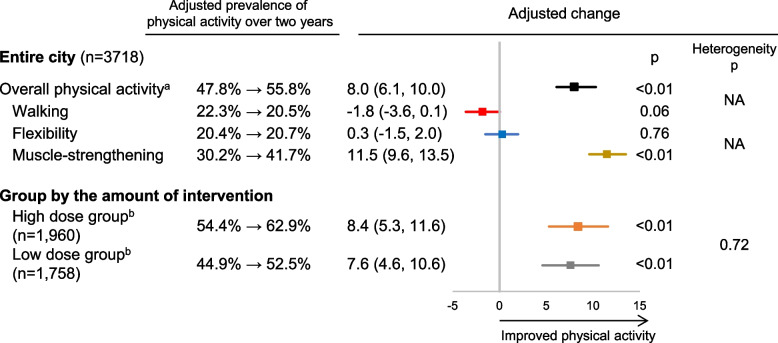
Effectiveness of a two-year citywide intervention on population-level physical activity. Estimates are percentage points with their 95% confidence intervals in parentheses; and they are adjusted for sex, age, body mass index, self-rated health, years of education, employment status, engagement in farming, chronic disease history, and community where respondents lived. An adjusted change difference greater than zero signifies that the intervention had a positive effect (favorable for physical activity). ^a^Engagement in regular aerobic, flexibility and/or muscle-strengthening activities. If respondents met any one of three following conditions, they were defined as “engaging in regular physical activity”: (i) ≥ 150 min/week of walking, (ii) daily flexibility activity or (iii) ≥ 2 days/week of muscle-strengthening activity. ^b^High dose group: top ten communities in the implementation score, based on the naturally differing intervention doses across 29 intervention communities. Low dose group: bottom19 communities. The details of the implementation score are described in Appendix Tables 1 and 2

For the effectiveness evaluation, eligible data were obtained from 3,718 adults at baseline (response rate 53.0%). At the two-year survey, 2,963 adults responded (79.7% follow-up rate, Fig. 1). Table 1 lists the characteristics of the respondents at baseline, and Table 2 presents the unadjusted distribution of the physical activity outcomes at baseline and at the two-year follow-up. In the randomly sampled population, the proportion of males was 50.4% (3,530/7,000), with an average age of 60.4 years, whereas the proportion of males was 47.8% (1,776/3,718) among respondents, with an average age of 62.0 years. At the baseline, 53.4% (1,798/3,366) of the adults engaged in regular physical activity, and the proportion increased to 59.9% (1,647/2,751) two years later. The adjusted physical activity prevalence and amount of change are shown in Fig. 2. The adjusted change of the primary outcome was + 8.0% points (95% confidence interval [CI]: 6.1, 10.0), indicating a significant increase. For the specific physical activity, we observed a significant increase only for the muscle-strengthening activity (adjusted change: + 11.5% points [95% CI: 9.6, 13.5]; walking: −1.8% points [95% CI: −3.6, 0.1]; flexibility activity: + 0.3% points [95% CI: −1.5, 2.0]).

**Table 1:** Baseline characteristics of the communities and participants (2016, Unnan City)

	Entire city	High dose group^a^	Low dose group^a^
Community	29	10	19
Residents, *n*	40,372	21,246	19,126
Residents aged 40–79 years, *n*	20,830	10,940	9890
Population density, mean (SD), /km^2^	73.0	135.6	48.2
Evaluation participations (eligible response rate)	3718/7000 (53.0%)	1960/3847 (50.9%)	1758/3153 (55.8%)
Male	1776 (47.8%)	923 (47.1%)	853 (48.5%)
Age, mean (SD), years	62.0 (10.2)	61.7 (10.4)	62.3 (10.0)
40–59 years	1370 (36.8%)	722 (36.8%)	648 (36.9%)
60–79 years	2348 (63.2%)	1238 (63.2%)	1110 (63.1%)
Body mass index, mean (SD), kg/m^2^	22.5 (3.1)	22.6 (3.1)	22.5 (3.2)
< 18.5	285 (7.8%)	153 (7.9%)	132 (7.6%)
≧18.5, < 25	2683 (73.1%)	1405 (72.6%)	1278 (73.7%)
≧25	701 (19.1%)	377 (19.5%)	324 (18.7%)
Self-rated health			
Excellent/good	3130 (86.2%)	1639 (85.6%)	1491 (86.8%)
Fair/poor	501 (13.8%)	275 (14.4%)	226 (13.2%)
Years of education, mean (SD)	12.0 (2.3)	12.2 (2.3)	11.7 (2.3)
Employed	2402 (66.1%)	1249 (65.0%)	1153 (67.4%)
Engagement in farming	1880 (51.8%)	849 (44.7%)	1031 (59.6%)
Chronic disease history^b^	2209 (59.4%)	1170 (59.7%)	1039 (59.1%)

*SD* standard deviation. Figures are n (%), n/N (%) or mean (SD) before imputation of missing values unless stated otherwise. Sample sizes (denominators) vary due to missing values

^a^High dose group: top ten communities in the implementation score, based on the naturally differing intervention doses across 29 intervention communities. Low dose group: bottom 19 communities. The details of the implementation score are described in Appendix Tables 1 and 2

^b^Having the following disease history: hypertension, hyperlipidemia, diabetes, hyperuricemia, cerebrovascular disease, heart disease, kidney and urologic diseases, liver disease, gastrointestinal disease, endocrine disease, cancer

**Table 2:** Distribution of physical activity at baseline and 2-year follow-up (2016–2018, Unnan City)

	Entire city (*n* = 3718)	High dose group^a^ (*n* = 1960)	Low dose group^a^ (*n* = 1758)	ICC^b^
**Overall regular physical activity**^**c**^**, *n***				
At baseline	1798 (53.4%)	941 (52.8%)	857 (54.2%)	0.0053
At 2 years	1647 (59.9%)	866 (59.6%)	781 (60.2%)	
**Total walking time, min/week**				
Median (IQR) at baseline	30 (0–150)	516 (29.8%)	386 (25.6%)	0.0055
Median (IQR) at 2 years	40 (0–170)	417 (29.7%)	367 (29.3%)	
≥ 150, *n* at baseline	902 (27.8%)	35 (0 – 170)	30 (0 – 140)	0.0029
≥ 150, *n* at 2 years	784 (29.5%)	40 (0 – 170)	30 (0 – 170)	
**Flexibility activity daily, *n***				
At baseline	784 (21.6%)	399 (20.8%)	385 (22.5%)	0.0046
At 2 years	642 (21.8%)	337 (21.8%)	305 (21.2%)	
**Muscle-strengthening activity, days/week**				
Median (IQR) at baseline	0 (0–3)	566 (30.0%)	528 (31.3%)	0.0054
Median (IQR) at 2 years	0 (0–4)	600 (40.4%)	528 (40.0%)	
≥ 2, *n* at baseline	1094 (30.6%)	0 (0 – 2.3)	0 (0 – 3)	0.0076
≥ 2, *n* at 2 years	1128 (40.2%)	0 (0 – 4)	0 (0 – 3)	

*SD* standard deviation. Figures are n (%), n/N (%) or mean (SD) before imputation of missing values unless stated otherwise. Sample sizes (denominators) vary due to missing values

^a^High dose group: top ten communities in the implementation score, based on the naturally differing intervention doses across 29 intervention communities. Low dose group: bottom 19 communities. The details of the implementation score are described in Appendix Tables 1 and 2

^b^ICC (Intra-cluster correlation coefficient) is a measure of the similarity of data within community. It was calculated as follows using data at the 2-year follow-up before multiple imputation: ICC = (BMS – WMS) / (BMS + [K – 1] WMS). BMS is the between mean square and WMS is the within mean square calculated by one-way ANOVA with community as a factor, and K is the mean number of samples by community

^c^Engagement in regular aerobic, flexibility and/or muscle-strengthening activities. If respondents met any one of the following three conditions, the respondents were defined as “engaging in regular physical activity”: (i) engaging in 150 min/week or more of walking, (ii) engaging in daily flexibility activity or (iii) engaging two or more days/week in muscle-strengthening activities

The subgroup analysis showed that physical activity increased in all the subgroups (Appendix Fig. 3). Additionally, a cross-sectional analysis of the subgroups, which were divided into communities that did and did not undergo the intervention in the cRCT stage of this study (2009–2014), showed a non-significant adjusted difference in the prevalence of regular physical activity in 2016: 1.9% points (95% CI: −1.5, 5.2) higher in nine intervention communities (48.6% [45.0, 52.1]), compared with three control communities (46.7% [42.4, 51.0]).

In the exploratory analysis of the dose–response relation between the amount of intervention and population-level change in physical activity, 10 communities (*n* = 1,960) were classified as the high dose group and 19 communities (*n* = 1,758) as the low dose group. Table 1 lists each group’s respondent characteristics, and Table 2 presents the unadjusted distribution of physical activity in both groups. When comparing the two groups, the low dose group was more likely to engage in farming and had fewer years of education. No group differences were observed for the other characteristics. The adjusted change in regular physical activity was slightly greater in the high dose group (+ 8.4% points [95% CI: 5.3, 11.6]), compared with the low dose group (+ 7.6% points [95% CI: 4.6, 10.6]), though not statistically significant change difference (P for heterogeneity = 0.72, Fig. 2).

Regarding the assessment of the attrition bias, the baseline physical activity participation rate among those who had missing values for the physical activity outcome at the two-year follow-up was 52.3% (432/826), which was similar to the participation rate of all the participants, totaling 53.4% (Appendix Table 5). Attrition bias alone may not fully explain the observed increase in regular physical activity. However, the result of the dose–response analysis could be affected by a slightly higher baseline physical activity participation rate among those with missing values at the two-year follow-up in the low dose group (54.5%), compared with that in the high dose group (50.4%); this difference was not statistically significant (*P* = 0.24).

## Discussion

The citywide scaled-up intervention increased the percentage of residents engaging in regular physical activity over two years. By the physical activity type, muscle-strengthening activity had a significant increase, contributing to the overall rise in physical activity. The predecessor to this study, the COMMUNICATE study, was the first cRCT that provided evidence for the effectiveness of a community-wide intervention in increasing physical activity at the community level [6, 10]. The present study further corroborates the effectiveness of broadening the reach of this established intervention method.

The COMMUNICATE study (cRCT) reported a 2.5% point increase in regular physical activity in the intervention group over five years [10]. In comparison, the present study observed a larger adjusted change of 8.0% points in the entire city after two years. This significant difference may be attributed to three key factors. First, the long-term initiatives of Unnan City may have played a crucial role. Since the initiation of the cRCT in 2009, Unnan City Hall and other collaborating organizations have been accumulating valuable expertise and intervention resources, such as promotional materials and human networks. Collectively, these resources may have contributed to the efficient rise in population-level physical activity. The second factor pertains to the intervention’s multi-level approach, aligning with the ecological model [7, 8]. This encompassed individual-level information provision, interpersonal-level training of local volunteers, and the establishment of the exercise facility at the environmental level. These diverse elements, openly integrated into the entire-city intervention but excluded from the cRCT design owing to contamination concerns, could have positively influenced the outcomes. The third factor relates to external influences, specifically, the general trend in muscle-strengthening activity. Nationwide surveys conducted by the Sasakawa Sports Foundation showed an increase in the percentage of individuals engaging in muscle-strengthening activity across all age groups, increasing from 13.7% in 2016 to 15.2% in 2018 [27, 28]. This indicates a growing popularity of muscle-strengthening activity. Our study did not quantify the individual contributions of the intervention elements and external factors to the promotion of physical activity. Future research should involve surveys, interviews, and exploration of the connection between the awareness of intervention components and physical activity. It should identify effective elements and areas for improvement in future initiatives.

The implementation evaluation revealed some strengths and weaknesses of this scale-up intervention. Although all the communities adopted implementation, the global implementation score was lower than that in the cRCT presumably because of the differences in the duration (2 vs 5 years) and the number of communities covered (29 vs 9). In addition, the implementation doses varied across communities, which could be attributed in part to the fact that the contents and frequency of the programs were determined by collaborating institutions rather than the Unnan City government alone. This could also have resulted in different changes in the three types of physical activities. While all the three types of physical activities were encouraged locally in various communities, there was a stronger citywide promotion for exercises integrating muscle-strengthening activity (e.g., the Unnan *Kou-un* exercise), driven by collaboration outcomes rather than intentional emphasis. Conversely, the modest walking promotion may not have effectively countered age-related declines [29]. Implementers are widely recognized as a key factor in successful implementation [30]. In the dissemination study of CHAMPS II, an individual-level, research-based physical activity promotion program in the US, the observed physical activity change was smaller than in the pre-trial phase. The authors noted that some components were lost in real-world settings, highlighting the importance of securing committed and adaptable personnel [31]. Similarly, Australia’s scaled-up school-based intervention showed smaller effect sizes compared to earlier trial, due to factors such as modifications in delivery and low fidelity; adequate support and minimum guidelines for schools were identified as necessary moving forward [32]. Although similar challenges may apply to this study, given that this is a community-wide intervention aimed at changing broader context [33], alternative approaches beyond focusing solely on the quantity or quality of implementers should be considered. One such approach, which emerged during collaboration in this study, involved physical environment interventions. However, the availability of recreational facilities such as gyms demonstrated a negligible correlation with walking behavior [34], indicating that the health promotion center had a limited impact on the walking behaviors in this study. The diversity of intervention channels and potential (collaborative) implementers is a strength of the community-wide intervention that needs to be leveraged; however, we need to be mindful of the potential for increased uncertainty in the quality and quantity of implementation in this type of large-scale interventions. Additionally, the overall variations in the intervention dose did not correspond to the presence or absence of intervention in the previous cRCT, suggesting the potential for disseminating the intervention to new locations.

This study encompassed some of the key elements of implementation science [35] and demonstrated several strengths. First, it engaged various organizations including local government, businesses, and volunteer groups, making necessary adaptations for smooth scaled-up implementation. In a dissemination study of the Strong Women-Healthy Heart program in the US, program maintenance was hindered by leader turnover due to job changes or retirement [36]. The involvement of diverse organizations is crucial for multi-faceted implementation and program sustainability. Holding frequent stakeholder meetings and investing in resident volunteer training were notable strengths in this project. Second, despite employing a single-arm before–after comparison design, this study also integrated dose–response analysis as a quasi-experimental component. Many other implementation studies share similar designs [16, 37]. For example, the scale-up study of Go4Fun, an Australian childhood obesity intervention, conducted pre- and post-intervention assessments, including a secondary analysis comparing completers (≥ 75% attendance rate) with non-completers [38]. Third, this study comprehensively evaluated the intervention using the RE-AIM framework. While other scale-up interventions have utilized RE-AIM or the Centers for Disease Control and Prevention’s Physical Activity Evaluation Handbook, to evaluate projects [31, 36], many have only partially evaluated elements such as *Effectiveness* and *Reach* [16]. A multidimensional, comprehensive evaluation is crucial for understanding and enhancing the challenges faced in promotion projects. Last, as a factual reference for considering the transferability of the COMMUNICATE study to other areas, a similar community-wide program called the “Fujisawa Plus 10 (+ 10)” project, inspired by the COMMUNICATE study, was implemented in Fujisawa City, Kanagawa Prefecture [11]. This project also demonstrated the effective promotion of physical activity at the five-year mark from the baseline.

Nonetheless, this study had several limitations. First, owing to the single-arm before–after comparison design, the results may have been affected by various types of bias, although non-randomized designs are typically employed for studies on dissemination and implementation [37]. Second, repeated measurements of the same cohort may have caused attrition bias. However, we confirmed that the influence of the attrition bias was small. Third, the baseline response rate of 53.0% was relatively low (vs 73.6% in the cRCT [10]). The eligible answers may not represent the target population and their true prevalence of physical activity could be lower, limiting the generalizability of the findings. Fourth, the survey used a self-administered questionnaire; the participants’ answers may have been affected by recall bias. However, evaluating physical activity in a large population can be costly and infeasible, especially when using objective measures, such as an accelerometer, thus favoring questionnaire surveys [39]. There are many types of questionnaires that vary in length. For example, longer questionnaires, such as the Community Health Activities Model Program for Seniors (CHAMPS), provide a detailed assessment of the respondents’ physical activity with less bias [40]. However, shorter tools, such as the single-item measure, have also demonstrated acceptable validity, reliability, and responsiveness [41, 42]. Our questionnaire regarding the primary outcome used only four items, which is relatively short. This is because the focus was not on the overall physical activity, but on three specific types of physical activities promoted in the intervention. Targeting the specific activities related to the intervention could be a practical option, as a few questions can meet the stakeholders’ interest, which is crucial in real-world settings [43]. Nevertheless, designing a questionnaire tailored to each intervention limits the ability to compare findings across studies. Therefore, it can be valuable to include short, standardized measures, such as the single-item measure [41, 42]. The use of validity- and/or reliability-tested questionnaires was an advantage of this study. Finally, although this study mainly reports quantitative evaluation, qualitative evaluation on the experiences of participants or those involved in the intervention would have provided invaluable insights. These qualitative evaluations are planned to be reported after the primary evaluation period, i.e., six years after baseline.

## Conclusions

This study evaluated a scaled-up intervention following a cRCT of a multi-strategic community-wide program for promoting population-level physical activity. Over a two-year citywide initiative, the physical activity level, particularly in muscle-strengthening activity, increased among middle-aged and older adults. The intervention was adapted to include new efforts tailored to the local availability of local resources. The adaptation process actively involved diverse stakeholders, including residents. Alongside promoting diverse physical activities, including aerobic activity and other types other than muscle-strengthening activity, future studies should scrutinize the long-term sustainability and health impacts of this scaled-up approach. Given the limited existing knowledge on community-wide interventions for promoting physical activity, further implementation and evaluation across diverse regions are imperative.

## Supplementary Information

Supplementary Material 1. Appendix Text. Intervention components and implementation. Appendix Table 1. The criterion for the implementation score. Appendix Table 2. Distribution of the implementation score among the communities. Appendix Table 3. Implementation of information, education, and support delivery (2016–2018). Appendix Table 4. Implementation adaptations in accordance with the FRAME-IS. Appendix Table 5. Distribution of physical activity at baseline among those who had missing values at 2-year follow-up. Appendix Fig. 1. Some examples of materials used in the intervention. Appendix Fig. 2. Flow chart for the different dimensions of the RE-AIM framework applied to the scaled-up COMMUNICATE study: Unnan, Japan, 2016–2018. CI, Confidence interval. a The details of the implementation scores are explained in Appendix Table 3. Appendix Fig. 3. Changes in regular physical activity over two years in subgroups. Estimates are percentage points with their 95% confidence intervals in parentheses; and they are adjusted for sex, age, body mass index, self-rated health, years of education, employment status, engagement in farming, chronic disease history and community where respondents lived as fixed effects, and individuals as a random effect. An adjusted change difference greater than zero signifies that the intervention had a greater effect (favorable for physical activity) on one group compared with the other. Boldface indicates *P* < 0.05. Regular physical activity was defined as engagement in regular aerobic, flexibility and/or muscle-strengthening activities. If respondents met any one of three following conditions, they were defined as “engaging in regular physical activity”: (i) ≥ 150 min/week of walking, (ii) daily flexibility activity or (iii) ≥ 2 days/week of muscle-strengthening activity. *Community-level assignment in the original cluster randomized controlled trial (cRCT).

## Data Availability

The datasets used and/or analyzed in this study are available from the corresponding author upon reasonable request, in accordance with the regulations and permissions of Unnan City.

## Abbreviations

BMI: Body mass index
cRCT: Cluster randomized controlled trail
GLMM: Generalized linear mixed model
PA: Physical activity
