# Supplementary material for: Two-year scale-up dissemination study of a multi-strategic community-wide intervention promoting physical activity: a single-arm pre-post hybrid effectiveness-implementation trial
Source: Int J Behav Nutr Phys Act. 2024 Nov 25;21:131. doi: 10.1186/s12966-024-01684-6 (PMC11590483; doi:10.1186/s12966-024-01684-6)
Supplement: Supplementary file 1 — Supplementary Material 1. Appendix Text. Intervention components and implementation. Appendix Table 1. The criterion for the implementation score. Appendix Table 2. Distribution of the implementation score among the communities. Appendix Table 3. Implementation of information, education, and support delivery (2016–2018). Appendix Table 4. Implementation adaptations in accordance with the FRAME-IS. Appendix Table 5. Distribution of physical activity at baseline among those who had missing values at 2-year follow-up. Appendix Fig. 1. Some examples of materials used in the intervention. Appendix Fig. 2. Flow chart for the different dimensions of the RE-AIM framework applied to the scaled-up COMMUNICATE study: Unnan, Japan, 2016–2018. CI, Confidence interval. a The details of the implementation scores are explained in Appendix Table 3. Appendix Fig. 3. Changes in regular physical activity over two years in subgroups. Estimates are percentage points with their 95% confidence intervals in parentheses; and they are adjusted for sex, age, body mass index, self-rated health, years of education, employment status, engagement in farming, chronic disease history and community where respondents lived as fixed effects, and individuals as a random effect. An adjusted change difference greater than zero signifies that the intervention had a greater effect (favorable for physical activity) on one group compared with the other. Boldface indicates P < 0.05. Regular physical activity was defined as engagement in regular aerobic, flexibility and/or muscle-strengthening activities. If respondents met any one of three following conditions, they were defined as “engaging in regular physical activity”: (i) ≥ 150 min/week of walking, (ii) daily flexibility activity or (iii) ≥ 2 days/week of muscle-strengthening activity. *Community-level assignment in the original cluster randomized controlled trial (cRCT). [file 12966_2024_1684_MOESM1_ESM.pdf]

Supplementary appendix to the article:

Two-year scale-up dissemination study of a community-wide intervention promoting physical activity: a single-arm pre-post hybrid effectiveness-implementation trial

CONTENTS

Appendix Text

Intervention components and implementation

References for Appendix Text

Tables

Appendix Table 1. The criterion for the implementation score

Appendix Table 2. Distribution of the implementation score among the communities

Appendix Table 3. Implementation of information, education, and support delivery (2016-2018)

Appendix Table 4. Implementation adaptations in accordance with the FRAME-IS

Appendix Table 5. Distribution of physical activity at baseline among those who had missing values at 2-year follow-up

Figures

Appendix Figure 1. Some examples of materials used in the intervention

Appendix Figure 2. Flow chart for the different dimensions of the RE-AIM framework applied to the scaled-up COMMUNICATE study: Unnan, Japan, 2016–2018

Appendix Figure 3. Changes in regular physical activity over two years in subgroups

Appendix Text

Intervention components and implementation

Following the intervention in the cluster randomized trial (cRCT) [1], the intervention in this scale-up study also consisted of three components: information delivery, education, and support delivery. Some adaptations for each implementation were made for various reasons. The sections below describe each components' implementations. Appendix Table 4 documents adaptation according to the FRAME-IS, the Framework for Reporting Adaptations and Modifications to Evidence-based Implementation Strategies [2].

1) Information delivery. Information regarding physical activity was disseminated by the institutions of Unnan City and community organizations, following the approach used in the cRCT. This included distributing documents such as flyers and posters. In the current scale-up intervention, the same materials were used, but new media channels, cable television and city newsletters, were added. These channels were not used in the cRCT to avoid contamination; however, we introduced them in the present single-arm scale-up study to reach a wider population. The messages conveyed were not limited to addressing low back and knee pain exclusively, as seen in the cRCT (e.g., "Be active to cure your low back and knee pain!"). Instead, a variety of messages were disseminated in collaboration with partner groups and related projects. Examples of these materials are provided in Appendix Fig.1. The distribution frequency and total amount of these materials were not predetermined. Owing to the need for flexibility and responsiveness, Unnan City government was

responsible for providing necessary information as situations arose, co-produce materials with collaborators, and make every possible effort to use a variety of media channels in order to reach a broader audience.

2) Education. As in the cRCT, outreach health education programs were implemented. The frequency and contents of the programs were determined by collaborating institutions rather than the Unnan City government alone. The city government worked to create an environment that facilitates the implementation of physical activity promotion by providing guidance from physical activity professionals, supporting the implementation of physical fitness tests (individual results were printed and given on the spot), and training lay volunteers to serve as exercise leaders (details in the next paragraph). A new initiative, the Unnan *Kou-un* (Happiness in Unnan) Exercise, was introduced by the local government of Unnan City through the Health, Medical, and Long-term Care Coordination Office (referred to hereinafter as the Coordination Office) to further accelerate the prevention of frailty in the communities in 2017. This program was inspired by the nationally renowned "*Iki iki hyakusai taisou* (Lively Centenarian Exercise)," which originated in Kochi prefecture. During the program, participants perform muscle-strengthening training with weights around their wrists and ankles while singing familiar rhymes. To provide implementation support, the Coordination Office lends weights, exercise music CDs, and posters describing exercise instructions and music lyrics to groups of five or more individuals committed to exercising at least once a week for three or more months. Rehabilitation experts offer guidance during the initial three sessions and assess physical fitness (e.g.,

walking speed) at baseline and again after three months. Apart from these visits, group members conduct their classes independently. The Coordination Office also promoted the Unnan *Kou-un* Exercise by disseminating information via phone or the Internet to various community organizations.

3) Support delivery. The social support component (i.e., network intervention) in the cRCT was slightly modified for the scale-up. While the cRCT focused on influencers identified within communities, the scale-up intervention utilized the existing volunteer training system in the local government. This change was made to maintain the network intervention while ensuring the intervention could be sustained on a larger scale [3,4]. These volunteers referred to as community exercise leaders played a pivotal role in disseminating knowledge and techniques related to health and exercise through word-of-mouth or health classes, all while being active members of the community themselves. Since 2006, Unnan City has been conducting training sessions for them. Some of their activities include "*Mame na kaiwa* (health-promoting dialogue)" which entails daily conversations incorporating health and exercise-related knowledge (derived from the local Izumo dialect, where "*mame*" means healthy and energetic). To increase their promotional activities, exercise leaders were organized by community so that they could work together. Follow-up training sessions and regular meetings were held where they could keep learning and share knowledge, experiences, and discuss concerns. Additionally, as a new form of physical environmental support, the refurbishment of an existing swimming pool facility was completed in July 2018, reopening as the health promotion city center. The facility now includes a warm water pool and training room as a municipal project, and

health promotion classes targeting a wide range of clients, from children to older adults, have been held there. While this facility was not opened for the purposes of the research, its opening coincided with the commencement of city-wide interventions for this study, as various health and sports activities in Unnan City gained momentum.

The intervention's main provider was the local government of Unnan City (responsible department: Physical Education and Medicine Research Center UNNAN). The department's promotion team consists of one public health nurse and four professionals in physical activity or nutrition, including two researchers and two exercise instructors. Eligibility criteria for team members include some form of certification related to physical activity promotion or public health.

One researcher (JK) oversaw the overall implementation of the intervention and the collaborations with other departments or institutions. Another researcher assisted with these responsibilities, alternating (from TA to TG) during the intervention period due to fixed-term employment contracts. The other three staff members implemented intervention in all communities. Health education and encouragement were provided by all staff members. The former researchers (MK and TA) continued to offer assistance and contribute to overall intervention management after leaving the local government.

Workshops on the basic theory and practice of social marketing, including interview skills training, were held for the department staff. All staff members were aware of the key message of the campaign and had general knowledge of physical activity recommendations as well as of the benefits

and precautions of physical activity for musculoskeletal diseases. The members were trained in brief mass-presentation techniques (delivery of an encouraging speech and demonstration of each type of physical activity all within a ten minute period) using a common manuscript to ensure standardization of the intervention. Case report forms of all interventions by the department were reviewed by fellow members. The department held meetings to foster the understanding of the intervention and participated in meetings by other organizations to build stronger networks. In total, the department attended 39 meetings and communicated with the following organizations: the public health (as affiliation), education and sports, regional development departments, and safety and health committee of Unnan City Hall, and with the community’s self-administered organization, Senior Citizens' Club, health-related volunteers, schools, universities, clinics, and private sectors.

References for Appendix Text

[1] Kamada M, Kitayuguchi J, Abe T, Taguri M, Inoue S, Ishikawa Y, et al. Community-wide intervention and population-level physical activity: a 5-year cluster randomized trial. Intl J Epidemiol. 2018;47(2):642-53.

[2] Miller CJ, Barnett ML, Baumann AA, Gutner CA, Wiltsey-Stirman S. The FRAME-IS: a framework for documenting modifications to implementation strategies in healthcare. Implement Sci. 2021;16(1):36.

[3] Kamada M, Kitayuguchi J, Abe T, Taguri M, Inoue S, Ishikawa Y, et al. Community-wide

promotion of physical activity in middle-aged and older Japanese: a 3-year evaluation of a cluster randomized trial. Int J Behav Nutr Phys Act. 2015;12(82).

[4] Valente TW. Network interventions. Science. 2012;337(6090):49-53.

[5] Van Acker R, De Bourdeaudhuij I, De Cocker K, Klesges LM, Cardon G. The impact of disseminating the whole-community project '10,000 Steps': a RE-AIM analysis. BMC Public Health. 2011;11(3).

Appendix Table1. The criterion for the implementation score

Score	Health educational program		Unnan *Kou-un* Exercise	Community exercise leader			Health promotion center (pool and gym)
	Quasi-population coverage of participants[a]	Frequency	Number of groups implementing	Community resident population per leader[b]	Frequency of the programs	Quasi population coverage of participants in the leaders' programs[c]	Quasi population coverage of users[d]
**10**	≥30%	≥Once a week	≥ 5	≤150	≥Once a week	80-100%	≥10%
**9**	-	≥Once in two weeks	-	151-200	≥Once in two weeks	-	-
**8**	20-29%	-	4	201-250	-	-	5-9%
**7**	-	≥Once a month	-	251-300	≥Once a month	60-79%	-
**6**	10-19%	-	3	301-400	-	-	2.5-4%
**5**	-	≥Once in three months	-	401-500	≥Once in three months	40-59%	-
**4**	5-9%	-	2	501-700	-	-	1.25-2.4%
**3**	-	≥Once a half year	-	701-1000	≥Once a half year	20-39%	-
**2**	1-4%	-	1	1001-2000	-	10-19%	-1.24%
**1**	-	≤Three times in two years	-	≥2000	≤Three times in two years	1-9%	-
**0**	0%	None	None	No leader	None	0%	0%

The score criteria were decided through discussion with the intervention implementers (Unnan City personnel).

[a]Quasi-population coverage of participants= (the annual average gross number of participants in health educational programs) / (the community population aged 40-79). The population considered for calculation is limited to those aged 40-79, as almost all the participants fall within this age range.

[b]Community resident population per leader= (the community population) / (the annual average number of community exercise leaders)

[c]Quasi-population coverage of participants in the leaders' programs= (the annual average gross number of participants in health and exercise programs led by leaders) / (the community population)

[d]Quasi-population coverage of users of the health promotion center= (gross number of users) / (the town population). Usage data is aggregated at the town level (six in total, the larger geographic unit that encompasses the communities), making the specific breakdown by communities unavailable.

Appendix Table 2. Distribution of the implementation score among the communities

	Whole city (29 communities)	High dose group (10 communities)	Low dose group (19 communities)
Mean (SD)	8.9 (4.5)	14.4 (2.6)	6.0 (1.7)
Median (IQR)	7.5 (5.3-12.5)	14.3 (12.5-16.5)	6.5 (4.8-7.5)
Maximum	18.5	18.5	8.5
Minimum	2.3	10.3	2.3

SD, standard deviation; IQR, interquartile range.

The implementation score is the sum of four intervention items on Appendix Table1. The categorization of the communities, based on the naturally differing intervention doses across 29 intervention communities, was conducted post hoc as an implementation outcome of the program.

Appendix Table 3. Implementation of information, education, and support delivery (2016-2018)

	Whole city[a] (29 communities)	High dose group (10 communities)	Low dose group (19 communities)	**Score[b]** (Whole city)
**Information delivery**				**71**
Handouts (numbers distributed)	173	76	0	16
Flyers (numbers distributed)	13145	1147	25	100
Leaflets (numbers distributed)	356	218	38	100
Posters (numbers hung)	653	117	10	100
Banners (numbers placed)	35	15	20	100
Exercise CDs with narration (numbers distributed)	13	2	4	22
Local audio broadcasts (times audio messages broadcasted)	16	16	0	3
Cable TV broadcasts	(Two exercise-programs were broadcasted every day citywide.)			100
City newsletters (times distributed to all households)	10	NA	NA	100
**Education**				**77**
Times health educational programs[c] implemented<br>-Gross number of participants	200<br>3503	115<br>1904	19<br>282	90
Number of groups implementing Unnan *Kou-un* Exercise	17	12	4	40
Times participated in *Challenge Day*[d]	2	NA	NA	100
**Support delivery**				**85**
Total number of community exercise leaders	166	105	61	93
-The number of visits and conversations with residents[e]	83053	64507	18546	86
-The number of exercise classes in which they were involved<br>-Gross number of their participants	1421<br>17734	967<br>14234	454<br>3500	79
Number of users of new health promotion center[f]	885 (5 towns)	NA	NA	83

NA, not applicable.

[a]Due to interventions not solely based on communities, the sums of the amounts of interventions for the high and low dose groups are not necessarily the same as the total amount of intervention for the whole city.

[b]For each item, a score of 100 (full score) was assigned based on the implementation status as follows: 100 is the score if the intervention was implemented in all 29 communities; otherwise, the score was the proportion of communities that implemented the intervention item and also takes into account the implementation of non-community-based (cross-boundary) interventions. A score of 90 was assigned if not all communities implemented the intervention, but the number of non-community-based interventions implemented was large and the score exceeded 100. For the three components (information delivery, education, and support delivery), their respective scores were derived taking the average of the intervention items within each component. This implementation scoring method is based on previous studies [1, 5] and confirmed afterwards through discussion with the project implementers (Unnan City personnel).

[c]Health educational programs here refer to those held by the Physical Education and Medicine Research Center Unnan.

[d]Challenge day is a nationwide event held annually, led by the Sasakawa Sports Foundation. It involves friendly competition with municipalities of similar size, competing based on the percentage of residents participating in the day's sports events.

[e]Dedicated dialogue with residents, which refers to a daily conversation incorporating knowledge related to health and exercise. The leaders report how many times they had the dialogues in the last month.

[f]Usage data is aggregated at the town level (six in total), making the specific breakdown by communities unavailable. The implementation score was derived based on the number of towns within the city where residents utilized the facility, with a maximum score of 100 for all six towns.

Appendix Table 4. Implementation adaptations in accordance with the FRAME-IS

**Module 1**	
The EBP being implemented is:	The COMMUNICATE study (multi-strategic community-wide intervention)
The implementation strategy being modified is:	1. Information delivery<br>2. Education delivery: outreach health education program<br>3. Support delivery: development of social support via network, and providing material support
The modification(s) being made is/are:	1. New channels were utilized, and more flexible contents/messages were accepted<br>2. A new exercise class package was promoted with demand from the other department.<br>3. (i) Change in the methods of network intervention, utilizing the existing volunteer training system in the local government. (ii) Removal of some supporting materials (e.g. renting pedometers). (iii) Collaboration with a new facility, which was built by the local government and operated by the private sector.
The reason(s) for the modification(s) is/are:	1. To reach a broader audience.<br>2. To strengthen implementer network and utilize external movement.<br>3. (i) To improve sustainability (ii) The lack of the local needs. (iii) To strengthen the implementer network and utilize external movement.
**Module 2**	
What is modified?	- Content (details provided in Module 3)<br>- Context (setting, scale up from nine communities to 29 communities, i.e., city-wide promotion)
**Module 3**	
What is the nature of the content, evaluation, or training modification?	- Loosing structure (1. Message based on social marketing was relaxed)<br>- Adding elements (1, 2, 3(iii))<br>- Substituting (3(i))<br>- Removing/skipping elements (3(ii))
OPTIONAL: what is the relationship to core elements?	- Fidelity consistent (1, 2, 3)
**Module 4**	
What is the goal?	- Increase reach of the EBP. (1, 2, 3(iii))<br>- Increase sustainability of the EBP. (3(i))
What is the level of the rationale for the modification?	- Organizational and implementer level

**Module 5**	
When is the modification initiated?	- Scale up (from nine communities to 29 communities)
Is the modification planned?	- Planned/Proactive (1, 3(i))<br>- Unplanned/Reactive (2, 3(ii),(iii))
**Module 6**	
Who participates in the decision to modify?	- Program administrator/Implementer (The Unnan City government)
OPTIONAL: Who makes the ultimate decision?	- Program manager/Implementer (The Unnan City government (Physical Education and Medicine Research Center UNNAN; Health, Medical, and Long-term Care Coordination Office))
**Module 7**	
How widespread is the modification?	- Network system/community<br>- Implementation/facilitation team

Appendix Table 5. Distribution of physical activity at baseline among those who had missing values at 2-year follow-up

	Whole city (29 communities)	High dose group (10 communities)	Low dose group (19 communities)
**Follow-up rate**	2963/3718 (79.7%)	1569/1960 (80.1%)	1394/1758 (79.3%)
**Numbers of those who had missing values at 2-year follow-up, n (%)**			
Overall regular physical activity[a]	967 (26.0%)	507 (25.9%)	460 (26.2%)
Walking	1058 (28.5%)	554 (28.3%)	504 (28.7%)
Flexibility activity	778 (20.9%)	412 (21.0%)	366 (20.8%)
Muscle-strengthening activity	914 (24.6%)	476 (24.3%)	438 (24.9%)
**Baseline distribution of those with missing outcome values at 2-year follow-up, n (%)**			
Overall regular physical activity[a]	432 (52.3%)	218 (50.4%)	214 (54.5%)
Walking	215 (27.2%)	119 (28.3%)	96 (25.9%)
Flexibility activity	179 (19.2%)	81 (16.6%)	98 (22.1%)
Muscle-strengthening activity	244 (27.0%)	122 (25.6%)	122 (28.6%)

[a]Engagement in regular aerobic, flexibility and/or muscle-strengthening activities. If respondents met any one of the following three conditions, the respondents were defined as 'engaging in regular physical activity': (i) engaging in 150 min/week or more of walking, (ii) engaging in daily flexibility activity or (iii) engaging two or more days/week in muscle-strengthening activities.

a. Poster of exercise containing muscle-strengthening activities

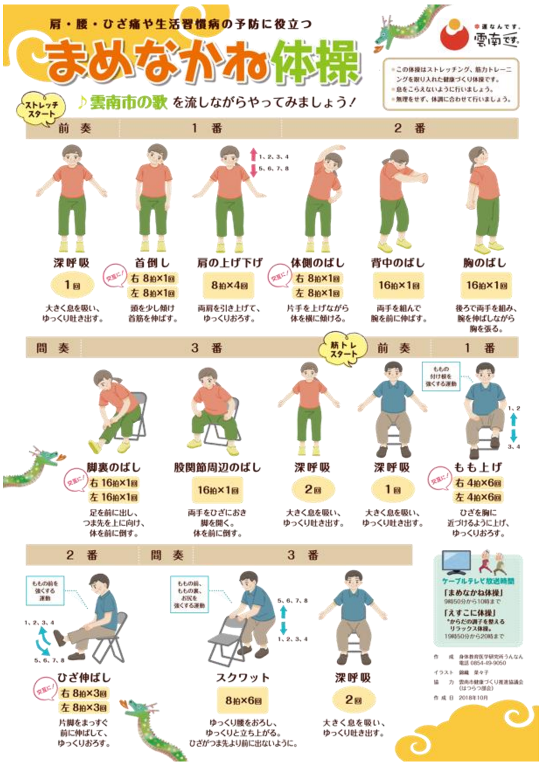

b. Flyer of exercise invented by a community organization

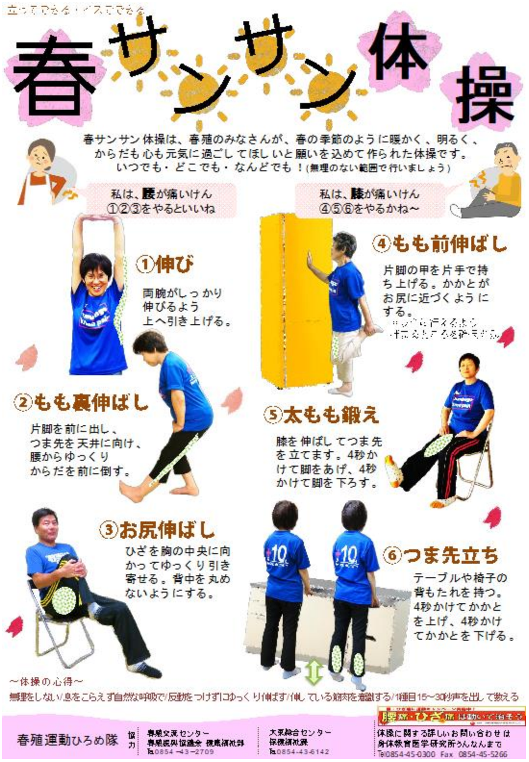

c. Leaflet about community exercise leaders

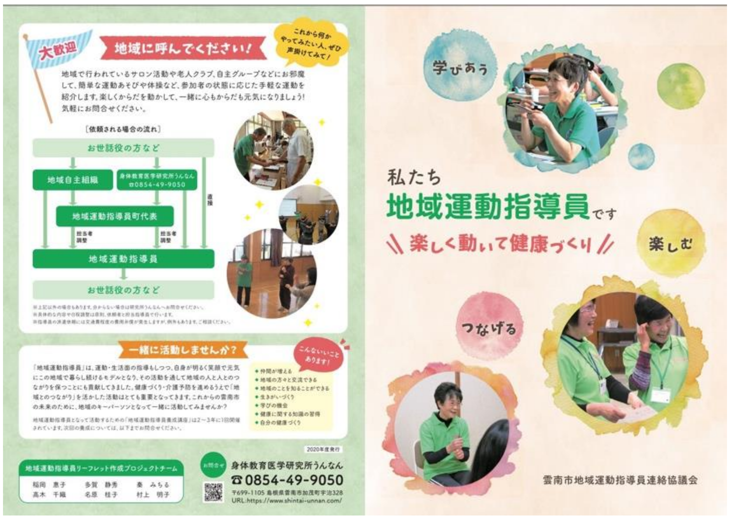

d. Blouson for walking event designed by a community organization

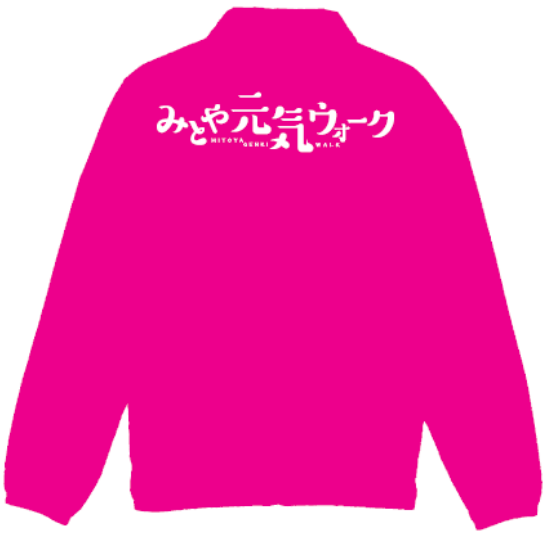

Appendix Figure 1. Some examples of materials used in the intervention

RE-AIM Issue

All 29 communities in Unnan city, Shimane prefecture, Japan

Adoption

Community that participated in the project: n=29 (**100%**)

Community that did not participate in the project: n=0 (0%)

Reach

Information was delivered to all households once with flyers, and 10 times with city newsletters ($Reach_1$=**100%**). Quasi-population coverage of education delivery: **10.6%** (2,215 gross number participation/ 20,831 adults living in the communities) in 2 years ($Reach_2$).

Implementation

Global implementation score: **78** on 100 (average of three intervention components; information delivery score of 71, education score of 77, and support delivery of 85) [a]

Efficacy/Effectiveness

Middle-age and older residents engaging in the recommended level of physical activity increased from 53.4% (1798/3366) to 59.9% (1647/2751) over 2 years (adjusted change: **8.0%pt** [95% confidence interval 6.1, 10.3]).

In high dose group (n=1,960 in 10 communities), those who engage in physical activity increased from 52.8% (941/1,784)→ 59.6% (866/1,453), adjusted change: 8.4%pt (95%CI: 5.3, 11.6)

In low dose group (n=1,758 in 19 communities), those who engage in physical activity increased from 54.2% (857/1,582) → 60.2% (781/1,298), adjusted change: 7.6%pt (95%CI: 4.6, 10.6)

Maintenance (setting level)

In 29 (**100%**) communities, the campaign has continued as of November 2022.

Maintenance (individual level)

NA (To be evaluated based on a follow-up survey conducted in November 2022.)

Appendix Figure 2. Flow chart for the different dimensions of the *RE-AIM* framework applied to the scaled-up COMMUNICATE study: Unnan, Japan, 2016–2018

CI, Confidence interval.

[a]The details of the implementation scores are explained in Appendix Table3.

Subgroups at baseline	n	Adjusted prevalence in regular physical activity at baseline (95%CI)	Adjusted change (95%CI)	Adjusted change difference (95%CI)
Female	1,942	50.3% (47.3, 53.4)	**+8.3%pt (5.3, 11.3)**	0.5%pt (-3.9, 5.0)
Male	1,776	45.3% (42.1, 48.4)	**+7.8%pt (4.8, 10.8)**	
40-59 years old	1,370	48.1% (43.9, 52.3)	**+7.2%pt (3.9, 10.5)**	1.4%pt (-2.8, 5.5)
60-79 years old	2,348	47.6% (44.3, 51.0)	**+8.6%pt (6.0, 11.1)**	
Excellent/good health	3,130	50.8% (48.4, 53.3)	**+7.6%pt (5.5, 9.7)**	3.4%pt (-2.3, 9.2)
Fair/poor health	501	43.5% (38.9, 48.1)	**+11.0%pt (5.6, 16.4)**	
Employed	2,402	43.9% (40.9, 46.8)	**+9.5%pt (7.0, 12.1)**	**4.4%pt (0.09, 8.6)**
Unemployed	1,233	52.4% (48.8, 56.1)	**+5.2%pt (1.9, 8.5)**	
Engagement in farming	1,880	49.5% (46.4, 52.6)	**+7.5%pt (4.8, 10.2)**	1.1%pt (-2.8, 5.1)
No engagement in farming	1,749	46.1% (42.8, 49.3)	**+8.6%pt (5.8, 11.5)**	
Chronic disease history	2,209	49.0% (46.0, 51.9)	**+7.1%pt (4.4, 9.9)**	2.2%pt (-2.1, 6.6)
No chronic disease history	1,509	46.4% (43.0, 49.8)	**+9.4%pt (6.2, 12.5)**	
Intervention in cRCT* (9 communities)	1,068	57.0% (41.7, 72.3)	**+7.9%pt (4.4, 11.4)**	2.6%pt (-4.4, 4.9)
Control/Excluded in cRCT* (20 communities)	1,895	44.1% (36.9, 51.3)	**+8.1%pt (5.5, 10.8)**	
Whole city	3,718	47.8% (45.1, 50.5)	**+8.0 (6.1, 10.3)**	

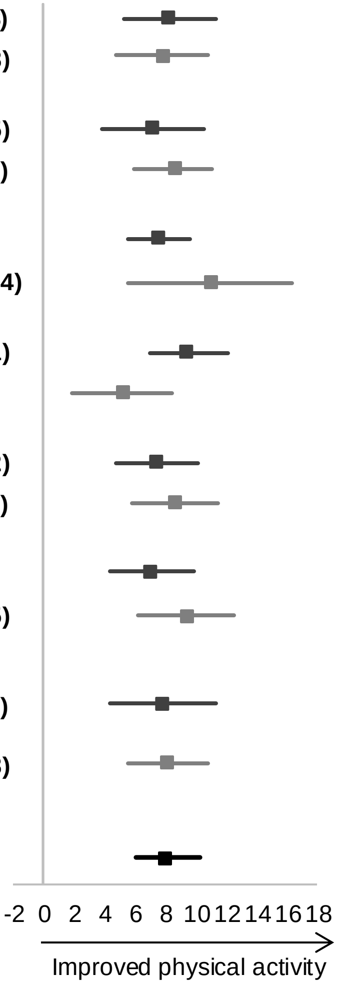

Appendix Figure 3. Changes in regular physical activity over two years in subgroups

Estimates are percentage points with their 95% confidence intervals (CI) in parentheses; and they are adjusted for sex, age, body mass index, self-rated health, years of education, employment status, engagement in farming, chronic disease history, and community where respondents lived as fixed effects, and individuals as a random effect. An adjusted change difference greater than zero signifies that the intervention had a greater effect (favorable for physical activity) on one group compared with the other. Boldface indicates P < 0.05. Regular physical activity was defined as engagement in regular aerobic, flexibility, and/or muscle-strengthening activities. If respondents met any one of three following conditions, they were defined as ‘engaging in regular physical activity’: (i) ≥150 mins/week of walking, (ii) daily flexibility activity, or (iii) ≥2 days/week of muscle-strengthening activity.

*Community-level assignment in the original cluster randomized controlled trial (cRCT)
